# Opioid harm reduction and stigma: proposed methods to improve the perception of people with addiction

**DOI:** 10.3389/fpsyt.2023.1197305

**Published:** 2023-08-10

**Authors:** Enrique López-Ramírez, Mary J. Huber, Diana Matías-Pérez, Gonzalo Santos-López, Iván Antonio García-Montalvo

**Affiliations:** ^1^División de Estudios de Posgrado e Investigación, Tecnológico Nacional de México/Instituto Tecnológico de Oaxaca, Oaxaca, Mexico; ^2^Clinical Rehabilitation Program, Wright State University, Dayton, OH, United States; ^3^Facultad de Medicina y Cirugía, Universidad Regional del Sureste, Oaxaca, Mexico; ^4^Departamento de Ingeniería en Desarrollo Comunitario, Tecnológico Nacional de México/Instituto Tecnológico del Valle de Etla, Oaxaca, Mexico

**Keywords:** stigma, addiction, harm reduction, violence, public drug policies

## 1. Introduction

In the last two decades, the opioid overdose crisis has attracted attention in the United States, and several treatments, prevention, and information programs have emerged, but despite this awareness, overdose death rates continue to increase (1). For example, in 2020, opioid-related overdose deaths reached 70,000, and by 2021, the number increased by 15% (2). The COVID-19 pandemic has resulted in a notable increase in drug overdose deaths, with 96,779 deaths occurring from March 2020 to March 2021 in the US alone (3). The devastating war on drugs has not produced results in terms of reducing drug consumption, nor has it succeeded in breaking up drug cartels. People who carry or consume drugs are criminalized, resulting in an estimated 65% of incarcerated people having a diagnosed substance use disorder (SUD) and 20% are incarcerated for drug-related crimes (4).

Unfortunately, many lives could be saved or rehabilitated if the medical field, the criminal system, and our cultural perspective incorporate the fact that SUD, and more specifically opioid use disorder (OUD) is a disease, not a moral failure (5, 6) and that there are many harm reduction techniques that are effective in saving lives. Both the disease itself and harm reduction techniques are stigmatized. SUD is a treatable chronic medical disease that involves complex interactions between brain circuits, genetics, the environment, and an individual's life experiences (7). The underpinning of this stigma is the scientifically unfounded belief that the taking of compulsive drugs by people with addiction reflects ongoing deliberate antisocial or deviant choices. This belief contributes to the continued criminalization of drug use and addiction (7). The following argument is based on evidence that supports the use of harm reduction techniques to reduce the staggering statistics above. We believe that the underuse of harm reduction techniques across the country can be primarily attributed to the stigma surrounding addiction.

## 2. Harm reduction

Harm reduction can be interpreted as any approach that aims to reduce risk, promote safety, and prevent disease or disability [(8) p. 50–51]. Two effective harm reduction techniques are medically assisted treatments (MAT) and safe injection sites (SIS). Effective MAT such as methadone, buprenorphine, and naltrexone reduce the use, overdose, and deaths from opioids. Importantly, they reduce the risk of infectious disease transmission and criminal behavior associated with drug use (9). These medications increase the likelihood that a person will remain in treatment, which itself is associated with a lower risk of overdose mortality, a reduced risk of Human Immunodeficiency Virus (HIV) and Hepatitis C Virus (HCV) transmission, reduced involvement in criminal justice, and a higher probability of employment (10). Unfortunately, the multiple and pervasive stigmas associated with OUD or SUD are formidable barriers to proven treatment (11).

There is a belief, primarily by those who believe in abstinence-based paradigms for those with OUD, that SIS promote drug use (12). There is an abundant amount of evidence to support SIS centers. The city of Vancouver, Canada, is a great example of connectedness between those with OUD and the community. For years, Vancouver has offered SIS where they offer clean needles, fentanyl test strips, and a safe place to inject drugs with medical care on site. The person injecting is not allowed to leave until they are free of an overdose. This model reduces criminal behavior, eliminates disease transfer, reduces overdoses, and eliminates deaths (13). The model has increased recovery rates among those who use the service due to the respect and empathy provided, as well as meeting the person in their recovery journey. Over time, the hope is that an individual moves through the stages of change to a place of contemplation, preparation, and action (14), ultimately becoming a functional and socially productive individual (15).

## 3. Stigmatization

There are those who consider harm reduction as a sign of defeat and consider harm reduction to be extreme, permissive, and enabling (16). The view that harm reduction enables and supports drug use continues to be based on a belief system that drug use is strictly a matter of individual choice and that external social and environmental factors are unrelated or unimportant in the analysis of why people use substances (17). This perception is completely oblivious to the fact that the American Society of Addiction Medicine (18) considers addiction a disease. Narratives in the media about personal experiences of treatment and recovery of people using opioids, including their positive messages about the effectiveness of treatment showcasing their success stories, could help reduce stigma (19).

## 4. Proposed methods to improve the perception of people with addiction

We adamantly believe that society, as a large community, can change the perception of addiction and harm reduction techniques. In terms of public policy, decision makers should follow the example of countries like Canada, in terms of the social integration offered to people with SUD who enter SIS. We recommend programs that help individuals learn new skills and competencies for daily work living, increasing the quality of life among those who learn new skills.

We implore public health policy makers to work hard to reduce stigmatizing attitudes among law enforcement, physicians, emergency room personnel, and others who work closely with people using opioids (20). Policy makers must create laws and policies that care for those with addiction vs. laws that punish them. Furthermore, policy makers must seek mechanisms that focus on positively informing communities about the benefits of the harm reduction approach through the provision of SIS and other techniques. These sites not only benefit people with SUD, but also have social benefits in terms of less illegal drug distribution on the street, a decrease in crimes related to the purchase and sale of drugs, and lower rates of blood-borne diseases, among others.

Social networks can play a positive role in conveying the message of the benefits of harm reduction. It is important that the results of scientific research on this approach can lead to a change from a focus on reporting the numbers of overdose deaths or deaths from drug-related crime to a focus on guiding consumers to seek SIS and the benefits that can be derived from them.

We believe that an ethical response to the opioid overdose crisis should include providing strong social support, removing social stigma and discrimination, and providing treatment and rehabilitation (21). The following are proposed methods to reduce stigma that surrounds addiction and harm reduction techniques.

### 4.1. Social justice

From a social justice approach, harm reduction is viewed as a loving practice guided by the principles of respect, acceptance, and dignity. It is based on the philosophy that every person should have the right to health and be treated with respect and compassion (22). This perspective focuses on maximizing the well-being of people with OUD; the belief is that people can recover (23). Health professionals can try to establish a trusting and supportive relationship with people who use a harm reduction service. Harm reduction should be seen as a treatment plan; as this treatment unfolds, success can be measured in various ways: longer periods of time between uses, better family or interpersonal relationships, decreased conflict with the law, ability to find and maintain employment, and also may be greater learning and self-efficacy (17).

### 4.2. Mass media campaign

Currently, there is a huge gap in terms of quality education and communication on the principles of harm reduction, stigma, and prevention of drug use (24). The government tries to justify its fight against drug use with zero tolerance and, second, with massive media campaigns. The infamous accountability to society forces them to report with figures how many prevention campaigns they launched in the media, to how many young people this information reached, or to launch sensational news about the fight against drug trafficking to justify that progress is being made in the field of prevention (25). As an example of this type of news, in 2016, the US Drug Enforcement Administration issued misinformation about the danger of exposure to fentanyl, warning that if touched, it could be absorbed by the skin (26). Substance use prevention programs should be designed using approaches focused on the development of strengths and social skills for conflict resolution and peace education. There is little to gain if prevention is focused solely on punishment, misinformation, repression, and stigmatization. We support a massive campaign, similar to the Reagan era of “just say no”, but a campaign focused on building resilience among young children and providing accurate education to adults.

### 4.3. Positive psychology and social networks

According to Nguyen et al. (27), people learn and reflect using information from social networks. Social networks could play a key role in reducing stigma toward people who use drugs (28). This approach comes from positive psychology, whose approach helps people combat negative environments through changes in mindset and behaviors (29). Therefore, programs focusing on positive and constructive aspects of harm reduction would be a good mechanism to draw the attention of public policy decision makers (30, 31).

From our perspective, it is necessary to resort to positive and constructive journalism, which can narrate the positive results reported in the scientific literature, in SIS, the experience of people in SUD treatment, and how they have managed to save their lives through timely administration of naloxone by a friend or close person who was trained to react and act in the face of possible overdose. Therefore, focusing on the positive and constructive aspects of the reduction of opioid harm would be a positive mechanism to attract the attention of public policy decision makers.

## 5. Education

The opioid overdose education and naloxone distribution programs (OEND) aim to reduce the risk of opioid death by facilitating educational training and increasing access to medications (32), which are also essential and effective in saving lives and reducing harm to individuals and communities (33).

Through education on the principles of the harm reduction approach, it is possible to inform the ways in which organizations, staff, and workers provide services, recognize the importance of the language used in this approach, learn about the strategies implemented, examine how culture and stigma can impact people who use drugs (34). We believe that if society is educated about addiction, more empathy and understanding can be generated toward those with addiction. Communities must understand that people experiencing addiction often face societal judgment, discrimination, and stigma that exacerbate the harm and violence in their lives (22); instead, they need care and treatment. As researchers and clinicians, we have learned that most people who start to use alcohol or drugs have suffered extreme trauma in their lives. Therefore, wanting to escape that pain can lead to addiction (35).

Schools must do their best to establish policies that are compassionate and forgiving, rather than punishing. Evidence has shown that students who experience exclusionary discipline are more likely to have problems with the justice system later. Instead of exclusion or punishment, schools must improve academic participation and decrease the number of excluded students for disciplinary reasons (36).

## 6. Conclusions

There is no need to invest more resources to determine whether harm reduction works or not. We firmly believe that it works. We think that the dollars set aside for testing harm reduction techniques should be used for implementing extensive programs. However, more scientific information is needed on how to implement positive and constructive information through the media that could change the perception of people in general and decision makers about MAT. It is also important to take into account the experience of consumers undergoing treatment and to distribute it in the media.

## Author contributions

All authors contributed to the design, development, and revision of the manuscript. All authors contributed to the article and approved the submitted version.
